# Supplementary material for: Nurses and grief: an analysis from the perspective of psychoanalysis
Source: Rev Bras Enferm. 2026 Sep 28;79:e7904. doi: 10.1590/0034-7167.20267904 (PMC13649212; doi:10.1590/0034-7167.20267904)
Supplement: Translation

# Translation: Enfermeiros e o luto: uma análise sob a perspectiva da psicanálise

**DOI:** 10.1590/0034-7167.20267904pt

**Authors:** Diane Maria Scherer Kuhn Lago, Jennifer Patrícia Kuhn Lago, Ana Claudia Afonso Valladares-Torres, Dirce Bellezi Guilhem

**Affiliations:** I Universidade de Brasília, Faculdade de Ciências e Tecnologias em Saúde. Ceilândia, Distrito Federal, Brasil; II Universidade de Brasília. Faculdade de Ciências da Saúde. Brasília, Distrito Federal, Brasil; III Arden University, MSc Psychology. Coventry, Reino Unido

A necessidade de compreender o luto no contexto atual do mundo, que enfrenta crises e desafios globais, como pandemias, guerras e violência social, evidencia a relevância deste estudo, pois a análise do luto, do processo de adaptação da pessoa enlutada, das formas de enfrentamento e do momento em que a ajuda profissional deve ser cogitada é importante para a saúde mental e o bem-estar subjetivo coletivo. Vários fatores influenciam a elaboração e vivência do luto, entre os quais se destacam as circunstâncias da perda sofrida pela pessoa. Conhecer os aspectos relacionados ao processo da perda ou da morte e do morrer é importante para o entendimento do luto^(1-4)^.

O luto pode ser definido como o sentimento desencadeado por perda significativa do elo entre uma pessoa e seu objeto. Representado por tristeza, foco na ausência e desmotivação, não se limita à morte, mas a perdas simbólicas ou reais ao longo do desenvolvimento da pessoa^(2,3)^.

Passar pela experiência de uma ruptura definitiva pode ser considerada traumática, principalmente pela circunstância da perda e pela importância da ausência do elo perdido^(1,4)^. Lacan^(2)^ descreve o luto como algo a ser superado de forma intersubjetiva, considerando que fatores externos, como os relacionamentos emocionais e o modo como a pessoa vive em sociedade, interferem na superação.

Considerando que o luto representa a dor da perda, o processo que o sucede representa a transformação da pessoa enlutada ao ressignificar a sua vida. Esse período é compreendido como um processo de elaboração e adaptação. Freud^(3)^ considera que o tempo supera a dor e que o luto, por ser um processo natural, não deve ter interferência. Porém, quando não evolui de forma natural, caracteriza-se como melancolia, um estado patológico de reação à perda.

Para que haja a superação do luto, é importante a ressignificação do que foi perdido. Não basta que o objeto ou a pessoa desapareça para que este seja esquecido; é necessário um trabalho psíquico lento e, muitas vezes, doloroso^(3)^. A perda pode causar dor, mas também crescimento e fortalecimento emocional. A resiliência e a capacidade de adaptação são fundamentais nesse processo^(4)^.

O luto, por ser um processo particular, envolve aspectos emocionais e psicológicos, como a capacidade de se reconectar com o mundo exterior, preencher o vazio da perda e ressignificar o presente e o futuro. Também envolve aspectos sociais de interação com o mundo exterior. Assim, quando ocorre a superação, o mundo interno da pessoa é modificado e a perda é ressignificada^(2,4)^.

Embora o processo seja singular, há momentos em que é possível perceber a necessidade de apoio profissional, especialmente da área da saúde mental, como o enfermeiro^(1)^. Reações comportamentais, cognitivas e emocionais variam, incluindo tristeza, raiva, culpa, desesperança e, em alguns casos, alívio^(1)^.

Quando o luto não é finito e há a sensação de que o objeto perdido representa também a morte de parte da pessoa enlutada, procurar um especialista é fundamental. Profissionais de saúde precisam estar preparados para identificar os sintomas do luto, compreender o processo e oferecer apoio no enfrentamento e no alívio do sofrimento^(3-4)^.

Os enfermeiros têm a missão de cuidar de forma integral, oferecendo suporte emocional, social e físico, além de conforto e atenção. Vivenciam perdas no cotidiano e são responsáveis pelo acompanhamento de pacientes e pessoas enlutadas, sendo essenciais nesse processo^(1)^. Portanto, há necessidade constante de qualificação para um cuidado eficaz, sem que o trabalho se torne fator de adoecimento. Pode-se concluir que a compreensão do luto na psicanálise e a discussão do processo de superação auxiliam esses profissionais de saúde a elaborar propostas de acompanhamento e apoio para pessoas enlutadas.

Com esta discussão, há maior conscientização sobre os sintomas do luto e os processos de enfrentamento, desde os considerados normais até os que podem representar quadros patológicos. A psicanálise não compreende o luto apenas como evento, mas também como uma forma de subjetivar a dor da perda, que pode ser constante. Quanto mais aprofundada a discussão, maior a compreensão sobre o processo de sofrimento e adaptação.

Enfim, reforça-se a importância de que enfermeiros e outros profissionais de saúde estejam atualizados e capacitados para esse atendimento, recebendo apoio social e psicológico para a oferta de acolhimento humanizado.

# Translation: Enfermeros y el duelo: un análisis desde la perspectiva del psicoanálisis

**DOI:** 10.1590/0034-7167.20267904es

**Authors:** Diane Maria Scherer Kuhn Lago, Jennifer Patrícia Kuhn Lago, Ana Claudia Afonso Valladares-Torres, Dirce Bellezi Guilhem

**Affiliations:** I Universidade de Brasília, Facultad de Ciencias y Tecnologías de la Salud. Ceilândia, Distrito Federal, Brasil; II Universidade de Brasília. Facultad de Ciencias de la Salud. Brasília, Distrito Federal, Brasil; III Arden University, MSc Psychology. Coventry, Reino Unido

La necesidad de comprender el duelo en el contexto actual del mundo, marcado por crisis y desafíos globales, como pandemias, guerras y violencia social, evidencia la relevancia de este estudio, ya que el análisis del duelo, del proceso de adaptación de la persona en duelo, de las formas de afrontamiento y del momento en que debe considerarse la ayuda profesional es importante para la salud mental y el bienestar subjetivo colectivo. Diversos factores influyen en la elaboración y vivencia del duelo, entre los cuales destacan las circunstancias de la pérdida sufrida por la persona. Conocer los aspectos relacionados con el proceso de pérdida, muerte y morir es importante para la comprensión del duelo^(1-4)^.

El duelo puede definirse como el sentimiento desencadenado por la pérdida significativa del vínculo entre una persona y su objeto. Representado por tristeza, foco en la ausencia y desmotivación, no se limita a la muerte, sino también a pérdidas simbólicas o reales a lo largo del desarrollo de la persona^(2,3)^.

Atravesar la experiencia de una ruptura definitiva puede considerarse traumático, principalmente debido a las circunstancias de la pérdida y a la importancia de la ausencia del vínculo perdido^(1,4)^. Lacan^(2)^ describe el duelo como algo que debe superarse de manera intersubjetiva, considerando que factores externos, como las relaciones emocionales y la forma en que la persona vive en sociedad, interfieren en dicho proceso de superación.

Considerando que el duelo representa el dolor de la pérdida, el proceso que le sucede representa la transformación de la persona en duelo al resignificar su vida. Este período se comprende como un proceso de elaboración y adaptación. Freud^(3)^ considera que el tiempo supera el dolor y que el duelo, por tratarse de un proceso natural, no debe sufrir interferencias. Sin embargo, cuando no evoluciona de forma natural, se caracteriza como melancolía, un estado patológico de reacción ante la pérdida.

Para que exista superación del duelo, es importante la resignificación de aquello que se perdió. No basta con que el objeto o la persona desaparezcan para que sean olvidados; es necesario un trabajo psíquico lento y, muchas veces, doloroso^(3)^. La pérdida puede causar dolor, pero también crecimiento y fortalecimiento emocional. La resiliencia y la capacidad de adaptación son fundamentales en este proceso^(4)^.

El duelo, por tratarse de un proceso particular, involucra aspectos emocionales y psicológicos, como la capacidad de reconectarse con el mundo exterior, llenar el vacío de la pérdida y resignificar el presente y el futuro. También involucra aspectos sociales relacionados con la interacción con el mundo exterior. Así, cuando ocurre la superación, el mundo interno de la persona se modifica y la pérdida es resignificada^(2,4)^.

Aunque el proceso es singular, existen momentos en que es posible percibir la necesidad de apoyo profesional, especialmente en el área de la salud mental, como el proporcionado por el enfermero^(1)^. Las reacciones conductuales, cognitivas y emocionales varían e incluyen tristeza, ira, culpa, desesperanza y, en algunos casos, alivio^(1)^.

Cuando el duelo no es finito y existe la sensación de que el objeto perdido representa también la muerte de una parte de la persona en duelo, buscar ayuda especializada resulta fundamental. Los profesionales de la salud necesitan estar preparados para identificar los síntomas del duelo, comprender el proceso y ofrecer apoyo en el afrontamiento y alivio del sufrimiento^(3-4)^.

Los enfermeros tienen la misión de brindar un cuidado integral, ofreciendo apoyo emocional, social y físico, además de confort y atención. Vivencian pérdidas en su práctica cotidiana y son responsables del acompañamiento de pacientes y personas en duelo, siendo esenciales en este proceso^(1)^. Por lo tanto, existe una necesidad constante de capacitación para un cuidado eficaz, sin que el trabajo se convierta en un factor de enfermedad. Puede concluirse que la comprensión del duelo desde el psicoanálisis y la discusión del proceso de superación ayudan a estos profesionales de la salud a elaborar propuestas de acompañamiento y apoyo dirigidas a personas en duelo.

Con esta discusión, existe una mayor concientización sobre los síntomas del duelo y los procesos de afrontamiento, desde aquellos considerados normales hasta los que pueden representar cuadros patológicos. El psicoanálisis no comprende el duelo únicamente como un evento, sino también como una forma de subjetivar el dolor de la pérdida, que puede volverse constante. Cuanto más profunda sea la discusión, mayor será la comprensión sobre el proceso de sufrimiento y adaptación.

Finalmente, se refuerza la importancia de que los enfermeros y otros profesionales de la salud permanezcan actualizados y capacitados para esta atención, recibiendo apoyo social y psicológico para ofrecer una acogida humanizada.
